# Physical activity–induced myokine responses in major mammalian farm animal species: a mini-review

**DOI:** 10.3389/fvets.2026.1836969

**Published:** 2026-06-15

**Authors:** Annika Krause, Katharina Metzger, Birger Puppe, Claudia Kalbe

**Affiliations:** 1 Research Institute for Farm Animal Biology (FBN), Dummerstorf, Germany; 2 Behavioral Sciences, Faculty of Agricultural and Environmental Sciences, University of Rostock, Rostock, Germany

**Keywords:** animal welfare, exercise, farm animal, locomotion, myokine, physical activity

## Abstract

Physical activity, including related concepts such as locomotion, movement, and exercise, is a fundamental behavior in humans and animals and contributes to physical and mental health. Although physical activity is essential for maintaining skeletal muscle function, farm animals are often kept under housing conditions with limited space and restricted opportunities for locomotion. Skeletal muscle secretes myokines that contribute to interorgan crosstalk; however, the relationship between physical activity and myokine-related responses in farm animals remains poorly understood. To summarize the current state of knowledge, we conducted a systematic literature search in PubMed, Scopus, and Web of Science in April 2026, focusing on studies addressing physical activity and myokine-related outcomes in major mammalian farm animal species. In total, 35 studies were identified. These studies examined several myokines, including interleukin-6 (IL6), fibronectin type III domain-containing protein 5 (FNDC5/irisin), brain-derived neurotrophic factor (BDNF), myostatin (MSTN), and insulin-like growth factor 1 (IGF1). The included studies showed substantial heterogeneity in experimental design, activity assessment, biological sample type, and the type, intensity, and quantification of activity. Overall, the current literature provides preliminary but fragmented evidence that physical activity is associated with myokine-related responses. However, a major limitation is the lack of standardized and objective methods for quantifying physical activity in farm animals, particularly within voluntary, self-directed activity paradigms. Conceptually, farm-relevant studies combining objective activity monitoring with skeletal muscle and systemic myokine analyses are required to clarify how physical activity contributes to muscle biology, performance, health, and welfare in livestock species.

## 1 Introduction

Skeletal muscle in farm animals is of particular interest because it constitutes the primary source of meat. Consequently, farm animals have undergone decades of selective breeding for enhanced muscle growth. In pigs, for example, approximately 50% of total body mass consists of muscle (1, 2). Although locomotion is essential for the proper development and function of skeletal muscle, farm animals are typically housed in confined systems that provide limited opportunities for species-appropriate locomotor behavior (3). The ability to express such behavior is fundamental not only for skeletal muscle development and maintenance but also for overall animal welfare. Locomotor behavior can provide insights into behavioral disorders and health status and has important welfare implications. However, studies quantifying distances covered by farm animals in conventional housing systems and at different developmental stages remain scarce (4, 5). In free-range systems, locomotion is often estimated indirectly, for example, based on the distance between feeding and resting areas (6, 7).

The terms “physical activity,” “locomotion,” “movement,” and “exercise” refer to related but distinct concepts in human and animal research, although they are often used interchangeably. Physical activity encompasses any bodily movement produced by skeletal muscles that results in energy expenditure, including locomotion and exercise (8). Locomotion specifically refers to motor activity that results in displacement of the whole body in external space (9). Exercise is a subset of physical activity that is planned, structured, and repetitive, with the objective of improving or maintaining physical fitness (8, 10). Under commercial housing conditions, farm animals have limited opportunities for physical activity, and exercise in the strict sense is virtually absent. In contrast, extensive human research has demonstrated that physical activity reduces the risk of cardiovascular and metabolic diseases, decreases the incidence of several cancers, enhances brain function, and alleviates pain and depression (11).

In recent years, several molecular mediators underlying the beneficial effects of exercise have been identified (12). These factors are collectively referred to as “exerkines,” defined as signaling molecules released in response to acute or chronic exercise that act via endocrine, paracrine, and/or autocrine pathways (13, 14). The concept that humoral factors mediate the systemic effects of exercise has long been recognized. Pedersen et al. demonstrated that contracting human skeletal muscle releases interleukin-6 (IL6) into the circulation during prolonged exercise, thereby introducing the term “myokine” (15, 16). Since then, additional myokines have been identified, including myostatin [MSTN; (17)], fibronectin type III domain-containing protein 5 [FNDC5, also known as irisin; (18)], insulin-like growth factor 1 [IGF1; (19)], and brain-derived neurotrophic factor [BDNF; (20)]. The biological relevance of myokines has been widely reviewed (21–25). More recently, the concept of the “myokinome,” encompassing the entirety of myokines, has provided a new framework for understanding how skeletal muscle communicates with other organs (26). In farm animals, however, knowledge about the role of myokines remains limited. Initial evidence in pigs suggests distance-related differences in myogenic growth potential (5), indicating that physical activity may influence muscle biology in livestock species.

This mini-review aims to provide a concise overview of current evidence on the effects of different forms of physical activity on myokine-related responses in major mammalian farm animal species, including pigs, cattle, goats, and sheep.

## 2 Methodology

**Figure 1:**
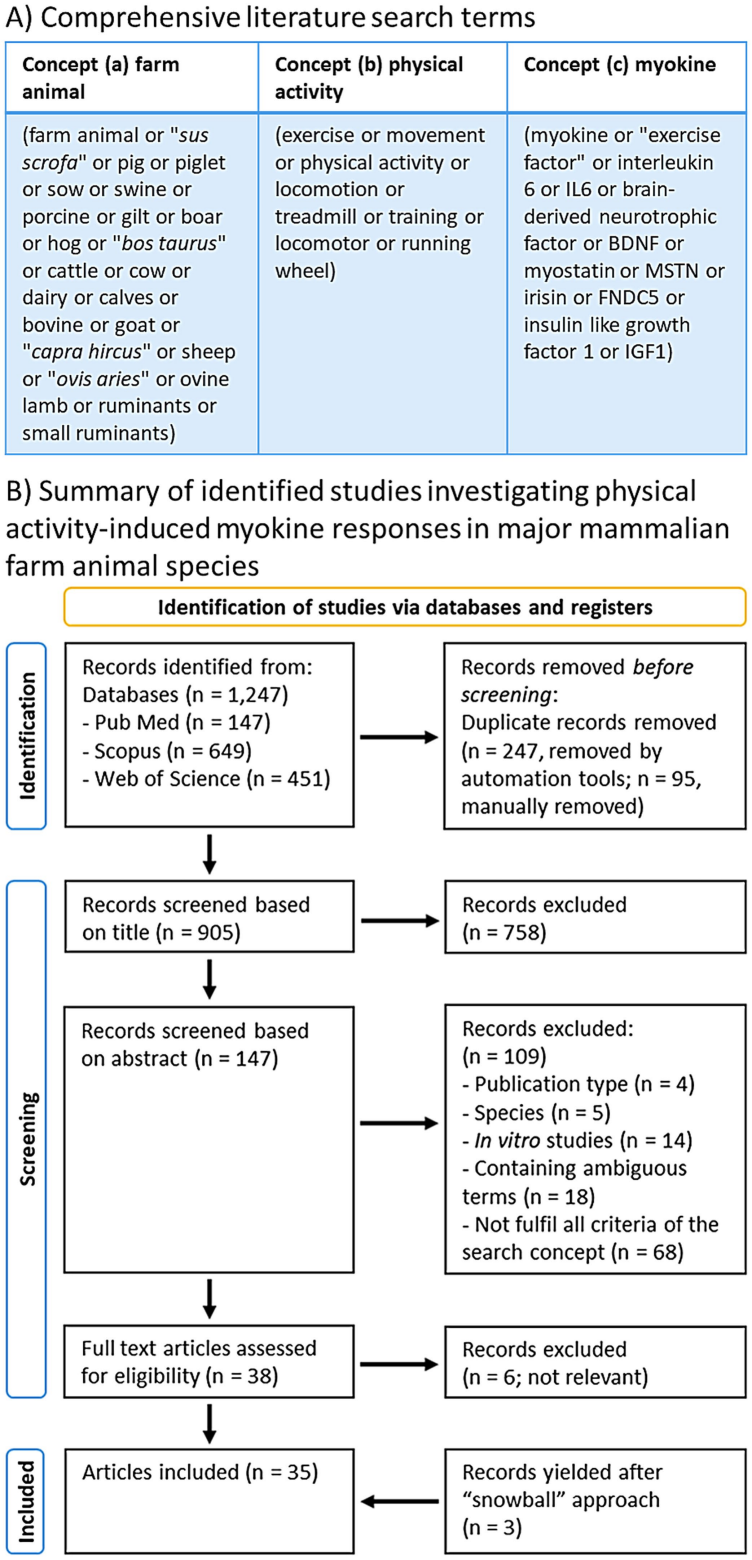
Search strategy and flowchart overview of record identification, screening, and final study selection. The diagram was adapted from the systematic review flow diagram template by Page et al. (77): grey boxes were removed as recommended, and the central section was modified to include our study-specific data.

The literature search was conducted in April 2026 and was broadly guided by the PRISMA guidelines [Preferred Reporting Items for Systematic Reviews and Meta-Analyses; (77)]. Searches were performed in PubMed, Scopus, and Web of Science using three search concepts: (a) “farm animal” and related terms, (b) “physical activity” and related terms, and (c) “myokine” and related terms (Figure 1A), following Jardat and Lansade (27). The full search strings are provided in the Supplementary material S1. Concepts were enclosed in parentheses and combined using the Boolean operator AND to retrieve publications addressing all three search concepts.

The farm animal concept included mammalian livestock species relevant to global red meat production, namely pigs, cattle, goats, and sheep (28). The physical activity concept included terms related to movement, locomotion, and exercise. The myokine concept included “myokine,” “exercise factor,” and specific myokines as defined by Severinsen and Pedersen (26), together with their official gene symbols (Supplementary material S2). Only English-language, peer-reviewed research articles were considered.

The article selection process is shown in Figure 1B. Briefly, duplicate records were removed before screening, either using the automated duplicate-detection tool in EndNote20 or manually (29). Titles were initially screened for publication type, language, and species. Abstracts of the remaining articles were then assessed, and records were excluded if they had an unsuitable publication type or species, described only *in vitro* studies, used ambiguous terminology unrelated to the search aims, or did not meet all search-concept criteria. The full texts of 38 articles were assessed for eligibility, of which six were excluded as irrelevant. The remaining 32 publications were subjected to a snowballing approach (30), which identified three additional studies and resulted in a final set of 35 articles.

## 3 Results

The species-specific analysis showed that studies addressing physical activity and myokine-related outcomes was identified in pigs [*n* = 18; (5, 31–37, 39–44, 55, 56, 60, 66)], followed by cattle [*n* = 13; (45, 47–54, 57–59, 61), sheep [*n* = 2; (46, 62)], and goats [*n* =  2; (38, 73)]. However, many studies were not originally designed as exercise- or activity-based myokine studies, but examined myokine-related markers within broader biomedical, nutritional, metabolic, reproductive, immune, or welfare contexts.

Across the identified studies, physical activity was assessed and quantified in markedly different ways (Table 1). Some studies objectively characterized the activity stimulus, whereas others used indirect proxies, such as housing conditions or environmental enrichment, or considered activity within frameworks in which it was not the primary exposure. Among studies that explicitly quantified physical activity—a key prerequisite for linking activity to potential myokine-related responses—two main methodological approaches were distinguished: forced exercise paradigms, marked in red in Table 1, and voluntary or spontaneous activity assessments, marked in green.

**Table 1:** Overview of studies investigating physical activity–induced effects on myokines in major mammalian farm animal species.

Physical activity	Quantification method	Research area	Farm animal species	Myokine	Refs.
Quantified					
Exercise training	Treadmill [same as 32]	Medical research/tissue metabolism	Pig*	FNDC5	(33)
Exercise training	Treadmill [45–85 min, 5 days/week, 16–20 weeks, 2.5–5 mph]	Medical research/cardiovascular disease	Pig*	IL6	(32)
Exercise training	Treadmill [same as 32]	Medical research/cardiovascular disease	Pig*	IL6	(36)
Exercise training	Treadmill [same as 32]	Medical research/cardiovascular disease	Pig*	IL6	(37)
Exercise training	Treadmill [30 min, 4 days/week, 4 weeks; warm-up, 3–4 km/h]	Medical research/osteoarthritis	Pig*	IL6	(35)
Exercise training	Treadmill [15 min, 2/day, 1.8 km/h]	Immunity/stress	Pig	IL6	(31)
Exercise training	Treadmill [85 min, 5 days/week, 16–20 weeks, 7–12 km/h, 8 km/day]	Medical research/cardiovascular. Disease	Pig*	IGF1	(34)
Exercise training	Treadmill [65–75 min, 5 days/week, 14 weeks, 4–6 mph]	Medical research/cardiovascular disease	Pig*	IGF1	(66)
Exercise training	Parcourse [50 days 7.5 km/day, *ø* speed 4.61 km/h]	Metabolism/nutrition	Goat	IGF1	(38)
Exercise training	Animal hot walker [20–90 min, 12 weeks, 2.5–4.5 mph]	Medical research/cardiovascular disease	Goat	IL6	(73)
Locomotor activity	Video-based VideoMotionTracker® distance [m/24 h]	Metabolism/animal welfare	Pig	BDNF, IGF1, MSTN	(5)
Walking	Video recorded [ethogram; proportion of time]	Animal welfare	Pig	IGF1, BDNF	(40)
Walking	Video recorded [ethogram; proportion of time]	Animal welfare	Pig	BDNF	(41)
Play behavior	Video recorded [ethogram; frequency]	Animal welfare/auditory enrichment	Pig [*]	IL6	(42)
General activity	Video-based motion detection PIGLwin software [% of time]	Neurodevelopment/inflammation	Pig	IL6	(43)
General activity	Video-based motion detection PIGLwin software [counts, %]	Neurodevelopment/behavior	Pig	IGF1	(39)
General activity	Live behavioral observation [ethogram, 10-min isolation test]	Immunity/stress	Sheep	IL6	(46)
Walking	Live behavioral observation [proportion of diurnal time]	Metabolism/nutrition	Cattle	IGF1	(45)
Targeted activity	Human-approach related activity [mean time to approach, s]	Metabolism/nutrition (LPS challenge)	Pig	IL6	(44)
Locomotor activity	Collar system daily activity [mov/h]	Reproduction/estrus detection	Cattle	IGF1	(48)
Locomotor activity	Pedometer [intensity (RI), duration (h)]	Reproduction/estrus detection	Cattle	IGF1	(50)
Locomotor activity	Pedometer [intensity index, duration (h)]	Reproduction/estrus detection	Cattle	IGF1	(51)
Locomotor activity	Pedometer [motion index, min/day, steps/day]	Metabolism/nutrition	Cattle	IGF1	(52)
Locomotor activity	Motion sensor [motion index, min/day, steps/day]	Metabolism/performance	Cattle	IGF1	(53)
Locomotor activity	Motion sensor [motion index, steps/day, laying bouts]	Metabolism/performance	Cattle	IGF1	(54)
Locomotor activity	Data logger [h/day]	Metabolism/nutrition	Cattle	IGF1	(47)
Locomotor activity	Data logger [time min/day; dur min/bout; freq bouts/day]	Immunity/heat stress	Cattle	IGF1	(49)
Without quantification					
Locomotor function	Scoring [1–5, no lameness—extreme]	Metabolism/nutrition	Cattle	IGF1	(57)
Locomotor function	Scoring [1–5; lameness—nonexistent]	Muscular development	Cattle	MSTN	(58)
Locomotor function	Porcine Thoracic Injury Behavior Scale [score 1–10]	Medical research/spinal cord injury	Pig*	IL6	(55)
Locomotor function	Porcine Thoracic Injury Behavior Scale [score 1–10]	Medical research/spinal cord injury	Pig*	IL6	(56)
Exhaustive exercise	No [max. 10 km in 3 h]	Metabolism	Cattle	MSTN	(59)
Indirect activity	No	Different housing systems	Cattle	BDNF, IL6	(61)
Indirect activity	No	Different housing systems	Sheep	IL6	(62)
Indirect activity	No	Animal welfare/environmental enrichment	Pig	BDNF	(60)

BDNF, brain-derived neurotrophic factor; FNDC5, fibronectin type III domain containing 5 (also known as irisin); IGF1, insulin-like growth factor 1; IL6, interleukin 6; MSTN, myostatin; Categorization based on voluntariness: red = forced, green = voluntary; Pig studies involving minipigs are marked with an asterisk*, hybrids between conventional pigs breeds and minipigs are marked with an asterisk in square brackets [*].

Forced exercise studies exposed animals to externally imposed, standardized activity protocols with predefined duration, intensity, frequency, and/or distance. This approach was used almost exclusively in porcine biomedical or translational exercise-training studies (31–37, 66). These designs are closest to classical exercise–myokine research in humans and rodents, and the identified treadmill studies therefore used minipigs as experimental models rather than conventional agricultural pig breeds. Their main advantage is the high degree of standardization and reproducibility, as key activity characteristics can be controlled. The treadmill-based studies investigated whether defined exercise was associated with changes in myokine-related, inflammatory, metabolic, vascular, or cardiovascular markers, including IL6- or IGF1-related outcomes. Among them, Fain et al. (33) provided the most direct activity–myokine approach by examining the FNDC5 pathway after exercise training. In goats, forced walking provided a defined locomotor stimulus, but did not clearly alter the investigated growth- and metabolism-related markers, including IGF-related pathways (38). Overall, forced physical activity studies in farm animal species approximate classical human and rodent exercise–myokine research, but evidence for direct activity-induced changes in myokine-related markers remains heterogeneous and context-dependent.

A second group of studies assessed voluntary or spontaneous physical activity within the animals’ housing environment. These studies are particularly relevant for farm-animal research because they capture self-motivated activity under conditions closer to routine production systems. However, they differed substantially in the precision and type of activity quantification. Some studies used video-based tracking, motion detection, or behavioral analysis to quantify distance covered, walking, play, exploration, locomotor activity, behavioral complexity, or time spent in active behaviors (5, 39–43). Others relied on direct behavioral observation, recording the occurrence or duration of behaviors such as walking, grazing, exploration, or approach behavior (44–46). A further group used objective individual-level measures based on sensors, accelerometers, pedometers, or data loggers, including step counts, walking time, motion indices, dynamic body acceleration, or posture changes (47–54). Despite these methodological differences, all approaches assessed activity under voluntary conditions, allowing animals to express self-motivated movement rather than being exposed to imposed exercise.

Most voluntary-activity studies did not assess classical circulating myokines as direct responses to physical activity. Instead, they investigated muscle-, metabolic-, reproductive-, immune-, or stress-related markers while including activity as a behavioral or physiological variable. Among these, Kalbe et al. (5) provided the most rigorous quantification of voluntary locomotor activity and indicated that the IGF2/MSTN mRNA ratio may serve as a sensitive molecular indicator of locomotor activity in pigs. Other video-based behavioral studies linked activity-related behaviors to growth factor, neurotrophic, immune, or developmental outcomes, including IGF1, BDNF-related expression, or IL6 (39–43). Together, these studies suggest that quantified voluntary activity in mammalian farm animals may be associated with molecular pathways involved in activity-dependent plasticity, neurodevelopment, or immune-related signaling. However, in most cases, metabolic hormones or immune/inflammatory markers, including IGF1 or IL6, were investigated within frameworks such as nutrition, stress, reproduction, or health, rather than as direct activity-induced myokine responses (44–54).

A further group of studies referred to physical activity or locomotion in a broader experimental context but did not quantify physical activity as a defined exposure. Several studies assessed locomotion only indirectly within forced-activity paradigms and were primarily designed as nutritional, medical, or rehabilitation-related investigations rather than as experimental activity studies (55–58). This includes the scoring of hindlimb movements in spinal cord injury models, where locomotion was evaluated mainly as part of functional recovery or rehabilitation rather than as a defined activity stimulus (55, 56). Another study focused more directly on muscle- and myokine-related signaling pathways, but did not directly measure physical activity; therefore, conclusions regarding activity-induced myokine regulation remain limited (59). In contrast, other studies used housing conditions or environmental modifications, such as increased space allowance, as indirect proxies for higher voluntary activity while assessing BDNF, IGF1, IL6, or MSTN-related pathways (60–62). In pigs, environmental enrichment appeared to be associated with increased serum BDNF level (60). However, because actual activity levels or distance covered were not objectively assessed, it remains unclear whether these effects were driven by enrichment *per se*, increased physical activity, or other environmental and behavioral factors.

Finally, myokine-related outcomes were measured in diverse biological compartments, including skeletal muscle, blood, adipose tissue, reproductive tissues, brain, intestine, vascular tissue, cerebrospinal fluid, and bone. However, only few studies assessed markers directly in skeletal muscle, the primary site of activity-induced myokine production, including FNDC5, MSTN, IGF-related transcripts, and BDNF (5, 33). Thus, most evidence reflects circulating or tissue-specific associations rather than direct muscle-derived myokine secretion in response to physical activity.

## 4 Discussion

This mini-review shows that evidence on physical activity and myokine-related responses in major mammalian farm animal species remains limited and heterogeneous. Although our systematic literature search identified 35 studies, only few were specifically designed to investigate myokine responses triggered by physical activity. Most studies assessed myokine-related markers within broader biomedical, nutritional, metabolic, reproductive, immune, or welfare contexts. Thus, the current literature provides indications of potential links between physical activity, muscle biology, and systemic signaling, but only limited evidence for direct muscle-derived myokine secretion.

Based on evidence from human and laboratory animal research, skeletal muscle is a key mediator of the physiological effects of physical activity (63). The concept that humoral factors mediate the systemic benefits of exercise is well established, and contracting muscle releases myokines, such as IL6, FNDC5, BDNF, and IGF1, thereby contributing to interorgan crosstalk (26, 64, 65). In the present mini-review, we focused on myokines well established in human exercise research and relevant to farm-animal studies. IL6 was the first myokine to be identified and remains one of the most intensively studied. However, it is also a cytokine associated with various disease states and is secreted by both skeletal muscle and activated immune cells (67, 68). MSTN is an inhibitor of muscle growth and is particularly relevant in farm animals because of its direct link to muscle development and carcass traits (17, 69). A potential functional counterpart to MSTN is IGF1, a key regulator of muscle metabolism (64, 65). IGF1 was frequently addressed in the reviewed studies, but was investigated in diverse contexts, including voluntary locomotion (5), forced walking (34), metabolism (53), neurodevelopment (39), and reproduction (48). FNDC5 represents another key exercise-associated factor, but was addressed only in the treadmill-trained pig study by Fain et al. (33). Both FNDC5 and BDNF are considered important components of a neuroprotective network (70). Although BDNF is expressed in skeletal muscle, it is not substantially released into the circulation (71); rather, it plays a pivotal role in linking physical activity to brain health and cognitive function (20, 72).

A central finding of this mini-review was the marked variation in how physical activity was defined and quantified. This variation limits study comparability and determines how observed myokine-related responses can be interpreted. Forced exercise studies, often conducted in minipigs rather than conventional agricultural pig breeds, provide standardized activity exposure and therefore most closely resemble classical exercise–myokine research in humans and rodents. These studies allow control over duration, intensity, frequency, and distance, and have examined inflammatory, metabolic, cardiovascular, regenerative, or growth-related outcomes, including FNDC5-, IL6-, and IGF-related markers (31, 32, 35–38, 66, 73). For example, Fain et al. (33) investigated FNDC5 responses in treadmill-trained pigs after 16–20 weeks of training, although exercise did not increase FNDC5 mRNA or protein expression in skeletal muscle. Although these approaches are methodologically robust and demonstrate the value of controlled activity exposure, their relevance for conventional livestock production is limited because the activity is externally imposed and typically involves biomedical models rather than agricultural breeds. In modern livestock production, increasing emphasis is placed on animal-friendly housing and welfare, including the ability of animals to express self-directed and self-motivated behavioral activity.

In contrast, voluntary-activity studies are more relevant for farm-animal research because they capture self-motivated locomotion under housing and management conditions closer to routine production systems. However, these studies varied widely in activity assessment, ranging from video tracking and behavioral observation to sensor-based measures, which limits direct comparability. A particularly strong example is the study by Kalbe et al. (5), in which voluntary locomotion in pigs was quantified at the individual level as walking distance using video tracking. This voluntary locomotor behavior influenced skeletal muscle expression of genes involved in myogenesis and muscle growth, including MSTN as a classical myokine, as well as the growth factor IGF2 and myogenic transcription factors such as MRF4 and MYOD (5). Thus, this study provides one of the clearest links between voluntary locomotor activity under conventional housing conditions and molecular adaptations in pig skeletal muscle.

Enrichment-related studies further quantified behavior using ethograms, including foraging, exploration, walking, play behavior, and inactivity. However, this approach captures the duration or proportion of behavioral categories rather than individual distance covered, step count, or speed (40, 41). In Brown et al. (41), enrichment-induced upregulation of genes related to neuronal activity and synaptic plasticity coincided with peaks in locomotion, consistent with findings in rodents suggesting reduced neuroinflammation and enhanced neuroprotection. In pigs, enrichment was associated with neurotrophic changes, including increased serum BDNF level and a tendency toward higher BDNF expression in the frontal cortex (40, 60). BDNF is essential for neuronal survival and plasticity, and elevated brain BDNF levels are associated with stress resilience (74). However, it remains unclear whether these effects result from enrichment itself, increased physical activity, greater distance covered, or other enrichment-related stimuli.

Sensor-based studies further demonstrate that voluntary activity can be assessed objectively and at the individual-animal level under practical farm conditions, at least in cattle. Data loggers have been used to quantify steps, motion index, lying and standing behavior, or movement activity, and these measures have been related to endocrine, metabolic, or physiological parameters, including IGF1 and health status. For example, IGF1 or IGF-related gene expression was measured in grazing, calf development, or reproductive contexts, where activity was related to grazing behavior or estrous expression rather than to a defined distance covered (47–52). Conceptually, these studies link farm-animal research to human exercise-myokine research through the role of IGF1 in growth, metabolic regulation, and muscle adaptation. However, IGF1 should be interpreted cautiously because circulating IGF1 reflects systemic endocrine regulation and is not necessarily a direct skeletal muscle-derived exercise signal (64). Thus, these studies show how activity and metabolic physiology can be assessed under practical farm conditions in cattle, but they were not designed to test whether voluntary physical activity directly induces myokine regulation.

Taken together, the current literature provides important methodological approaches, but not yet a coherent body of evidence. Forced-exercise studies provide a high degree of standardization because physical activity is clearly quantified, whereas voluntary-activity studies are more relevant for farm animals but often quantify physical activity less precisely or measure myokine-related factors in broader biological contexts. This indicates an important research gap. Future studies should combine objective, individual-level activity monitoring with targeted assessment of myokine-related markers in skeletal muscle and circulation. A promising approach would be to develop experimental behavioral paradigms in which animals can voluntarily engage in quantifiable physical activity, for example through self-initiated treadmill access or other operant locomotor challenges embedded in cognitive environmental enrichment. In domestic pigs, activation of intrinsic reward and motivational systems through cognitive enrichment has been shown to markedly increase voluntary behavioral activity, including quantified locomotor activity (75, 76). Conceptually, such approaches are particularly valuable in farm-animal research because they support the interpretation of physiological data in contexts more closely related to natural behavioral needs and animal welfare than to basic mechanistic research, as is often the case in laboratory and biomedical studies. Moreover, these approaches would preserve the voluntary nature of physical activity, while advanced video monitoring, automated AI-based behavioral analysis, and sensor-based technologies could quantify spontaneous locomotor activity under farm-relevant conditions. Reproducible and standardized protocols for behavioral activity assessment and physiological sampling, together with careful consideration of confounding factors such as breed, age, sex, housing system, nutrition, and health status, will be essential to distinguish direct effects of physical activity from broader environmental or management-related influences.

In conclusion, the current literature provides preliminary but fragmented evidence that physical activity is associated with myokine-related responses in mammalian livestock species. Forced-exercise studies offer a high degree of experimental and methodological control, but are largely limited to biomedical minipig models. In contrast, voluntary-activity studies are more relevant to livestock housing and welfare, but often lack precise activity quantification or direct evidence of muscle-derived myokine secretion. Clear methodological improvements are therefore needed in experimental design and analysis of physical activity as well as in the selection and measurement of myokines. Conceptually, farm-relevant studies combining objective activity monitoring with skeletal muscle and systemic myokine analyses are required to clarify how physical activity contributes to muscle biology, health, and welfare in livestock species.
