# Supplementary material for: Physical activity–induced myokine responses in major mammalian farm animal species: a mini-review
Source: Front Vet Sci. 2026 Jun 15;13:1836969. doi: 10.3389/fvets.2026.1836969 (PMC13312813; doi:10.3389/fvets.2026.1836969)
Supplement: Supplementary file 1 [file Table_1.DOCX]

**Physical activity–induced myokine responses in major mammalian farm animal species: a mini-review**

Supplementary Material

Annika Krause, Katharina Metzger, Birger Puppe, Claudia Kalbe

**S1 Search strings used for the online databases**

**PubMed (April 15, 2026; 10:46)**

For the advanced search in PubMed the following string was used:

(("farm animal"[Title/Abstract]) OR ("sus scrofa "[Title/Abstract]) OR ("pig"[Title/Abstract]) OR ("piglet"[Title/Abstract]) OR ("sow"[Title/Abstract]) OR ("swine"[Title/Abstract]) OR ("porcine"[Title/Abstract]) OR ("gilt"[Title/Abstract]) OR ("boar"[Title/Abstract]) OR ("hog"[Title/Abstract]) OR ("bos taurus"[Title/Abstract]) OR ("cattle"[Title/Abstract]) OR ("cow"[Title/Abstract]) OR ("dairy"[Title/Abstract]) OR ("calves"[Title/Abstract]) OR ("bovine"[Title/Abstract]) OR ("goat"[Title/Abstract]) OR ("capra hircus"[Title/Abstract]) OR ("sheep"[Title/Abstract]) OR ("ovis aries"[Title/Abstract]) OR ("lamb"[Title/Abstract]) OR ("ovine"[Title/Abstract]) OR ("ruminants"[Title/Abstract])) AND (("exercise"[Title/Abstract]) OR ("movement"[Title/Abstract]) OR ("physical activity"[Title/Abstract]) OR ("locomotion*"[Title/Abstract]) OR ("treadmill"[Title/Abstract]) OR ("training"[Title/Abstract]) OR ("locomotor"[Title/Abstract]) OR ("running wheel"[Title/Abstract])) AND (("myokine"[Title/Abstract]) OR ("exercise factor"[Title/Abstract]) OR ("interleukin 6"[Title/Abstract]) OR ("IL 6"[Title/Abstract]) OR ("IL-6"[Title/Abstract]) OR ("CDF"[Title/Abstract]) OR ("HGF"[Title/Abstract]) OR ("HSF"[Title/Abstract]) OR ("BSF2"[Title/Abstract]) OR ("BSF-2"[Title/Abstract]) OR ("IFNB2"[Title/Abstract]) OR ("IFN-beat-2"[Title/Abstract]) OR ("brain derived neurotrophic factor"[Title/Abstract]) OR ("BDNF"[Title/Abstract]) OR ("ANO2"[Title/Abstract]) OR ("BULN2"[Title/Abstract]) OR ("myostatin "[Title/Abstract]) OR ("MSTN"[Title/Abstract]) OR ("GDF8"[Title/Abstract]) OR ("GDF8:MSTN"[Title/Abstract]) OR ("GDF-8"[Title/Abstract]) OR ("MSLHP"[Title/Abstract]) OR ("irisin"[Title/Abstract]) OR ("fibronectin type III domain containing protein 5"[Title/Abstract]) OR ("FNDC5"[Title/Abstract]) OR ("FRCP2"[Title/Abstract]) OR ("insulin like growth factor 1"[Title/Abstract]) OR ("IGF1"[Title/Abstract]) OR ("IGF-1"[Title/Abstract]) OR ("IGF-I"[Title/Abstract]) OR ("IGFI"[Title/Abstract]) OR ("IGF"[Title/Abstract]) OR ("gIGFI"[Title/Abstract]) or ("Npt2B"[Title/Abstract]))

**Scopus (April 15, 2026; 10:42)**

For the advanced search in Scopus the following string was used:

TITLE-ABS-KEY (("farm animal" OR "sus scrofa" OR "pig" OR "piglet" OR "sow" OR "swine" OR "porcine" OR "gilt" OR "boar" OR "hog " OR "bos taurus" OR "cattle" OR "cow" OR "dairy" OR "calves" OR "bovine" OR "goat" OR "capra hircus" OR "sheep" OR "ovis aries" OR "lamb" OR "ovine" OR "ruminants") AND ("exercise" OR "movement" OR "physical activity" OR "locomotion" OR "treadmill" OR "training" OR "locomotor" OR "running wheel") AND ("myokine" OR "exercise factor" OR "interleukin 6" OR "IL6" OR "IL-6" OR "CDF" OR "HGF" OR "HSF" OR "BSF2" OR "BSF-2" OR "IFNB2" OR "IFN-beta-2" OR "brain derived neurotrophic factor" OR "BDNF" OR "ANON2" OR "BULN2" OR "myostatin" OR "MSTN" OR "GDF8" OR "GDF8:MSTN" OR "GDF-8" OR "MSLHP" OR "irisin" OR "fibronectin type III domain containing protein 5" OR "FNDC5" OR "FRCP2" OR "insulin like growth factor 1" OR "IGF1" OR "IGF-1" OR "IGF-I" OR "IGFI" OR "IGF" OR "gIGFI" OR "Npt2B"))

**Web of Science (April 15, 2026; 10:46)**

For the advanced search in Web of Science the following string was used:

TS=((farm animal or sus scrofa or pig or piglet or sow or swine or porcine or gilt or boar or hog or bos taurus or cattle or cow or dairy or calves or bovine or goat or capra hircus or sheep or ovis aries or lamb or ovine or ruminants) AND (exercise or movement or physical activity or locomotion* or treadmill or training or locomotor or running wheel) AND (myokine or "exercise factor" or interleukin 6 or IL6 or IL-6 or CDF or HGF or HSF or BSF2 or BSF-2 or IFNB2 or IFN-beta-2 or brain derived neurotrophic factor or BDNF or ANON2 or BULN2 or myostatin or MSTN or GDF8 or GDF8:MSTN or GDF-8 or MSLHP or irisin or fibronectin type III domain containing protein 5 or FNDC5 or FRCP2 or insulin like growth factor 1 or IGF1 or IGF-1 or IGF-I or IGFI or IGF or gIGFI or Npt2B ))

**S2** Official gene symbols related to major mammalian farm animal species and human

| **Official Symbol** | **Official Full Name** | **Species** | **Also known as** |
| --- | --- | --- | --- |
| BDNF | brain derived neurotrophic factor | *Sus scrofa* |  |
| BDNF | brain derived neurotrophic factor | *Bos taurus* |  |
| BDNF | brain derived neurotrophic factor | *Capra hircus* |  |
| BDNF | brain derived neurotrophic factor | *Ovis aries* |  |
| BDNF | brain derived neurotrophic factor | *Homo sapiens* | ANON2; BULN2 |
| FNDC5 | fibronectin type III domain containing 5 | *Sus scrofa* |  |
| FNDC5 | fibronectin type III domain containing 5 | *Bos taurus* |  |
| FNDC5 | fibronectin type III domain containing 5 | *Capra hircus* |  |
| FNDC5 | fibronectin type III domain containing 5 | *Ovis aries* |  |
| FNDC5 | fibronectin type III domain containing 5 | *Homo sapiens* | FRCP2; irisin |
| IGF1 | insulin like growth factor 1 | *Sus scrofa* | IGF-1; IGF-I; Npt2B |
| IGF1 | insulin like growth factor 1 | *Bos taurus* | IGF-1; IGF-I |
| IGF1 | insulin like growth factor 1 | *Capra hircus* | IGFI; IGF-1; IGF-I; gIGFI |
| IGF1 | insulin like growth factor 1 | *Ovis aries* | IGFI; IGF-1; IGF-I |
| IGF1 | insulin like growth factor 1 | *Homo sapiens* | IGF; MGF; IGFI; IGF-I |
| IL6 | interleukin 6 | *Sus scrofa* | IL-6 |
| IL6 | interleukin 6 | *Bos taurus* |  |
| IL6 | interleukin 6 | *Capra hircus* | IL-6 |
| IL6 | interleukin 6 | *Ovis aries* |  |
| IL6 | interleukin 6 | *Homo sapiens* | CDF; HGF; HSF; BSF2; IL-6; BSF-2; IFNB2; IFN-beta-2 |
| MSTN | myostatin | *Sus scrofa* | GDF8; GDF8:MSTN |
| MSTN | myostatin | *Bos taurus* | GDF8 |
| MSTN | myostatin | *Capra hircus* | GDF8; GDF-8 |
| MSTN | myostatin | *Ovis aries* | GDF8; GDF-8 |
| MSTN | myostatin | *Homo sapiens* | GDF8; MSLHP |

<https://www.ncbi.nlm.nih.gov/gene/> (April 10, 2026; 13:45)
